# A Performance-Based Teleintervention for Adults in the Chronic Stage after Acquired Brain Injury: An Exploratory Pilot Randomized Controlled Crossover Study

**DOI:** 10.3390/brainsci12020213

**Published:** 2022-02-03

**Authors:** Aviva Beit Yosef, Jeremy Michael Jacobs, Jeffrey Shames, Isabella Schwartz, Yafit Gilboa

**Affiliations:** 1 School of Occupational Therapy, Faculty of Medicine, Hebrew University of Jerusalem, Jerusalem 9124001, Israel; yafit.gilboa@mail.huji.ac.il; 2 Faculty of Medicine, Hebrew University of Jerusalem, Jerusalem 9124001, Israel; JacobsJ@hadassah.org.il (J.M.J.); IsabellaS@hadassah.org.il (I.S.); 3 Department of Geriatrics and Geriatric Rehabilitation, Hadassah Medical Center, Jerusalem 9124001, Israel; 4 Medical and Health Professions Division, Maccabi Health Services, Tel Aviv 6812509, Israel; Sheimes_j@mac.org.il; 5 Department of Rehabilitation and Physical Medicine, Hadassah Hebrew University Medical Center, Jerusalem 9124001, Israel

**Keywords:** chronic stroke, participation, neurorehabilitation, cognitive rehabilitation, telerehabilitation, occupation-based intervention, metacognitive strategy training, occupational therapy, self-efficacy, executive functions

## Abstract

This pilot study aimed to investigate the initial effect of a remotely delivered performance-based client-centered intervention on activity performance and participation among adults in the chronic phase after acquired brain injury (ABI). Sixteen participants living at home with little to no assistance in basic daily activities were allocated into intervention or waitlist control groups. Assessments were conducted at the baseline, after the 3-month intervention/wait period, and at a 3-month follow-up. The primary outcomes were activity performance using the Canadian Occupational Performance Measure (COPM) and the Performance Quality Rating Scale (PQRS) and participation using the Mayo-Portland Adaptability Inventory-4 (MPAI-4). The intervention included weekly videoconferencing sessions using the Cognitive Orientation to Daily Occupational Performance approach (tele-CO-OP). The participants identified five functional goals, of which three were directly addressed. Wilcoxon signed-ranks test results showed no significant improvements in the control group at the end of the 3-month wait period. Pooled data from both groups showed significant improvements in COPM scores for trained and untrained goals following the intervention. Significant improvements were also found in the PQRS and MPAI-4 scores. Improvements were partially maintained at follow-up. Our preliminary results suggest that tele-CO-OP may positively impact the lives of adults after ABI who are coping with long-term disability.

## 1. Introduction

Telerehabilitation has emerged as a promising service-delivery method to provide rehabilitation treatment for adults following acquired brain injury (ABI) living in the community [1,2,3,4]. It is defined as the use of information and communication technologies to provide remote rehabilitation services to people in their homes or other environments [5]. Telerehabilitation offers the advantage of enabling people to receive therapeutic services at home without having a therapist on site, thus increasing accessibility in rural areas and for those with limited access to transportation due to health conditions or socioeconomic factors. Another major advantage of this approach is that it allows us to observe how people perform their daily activities in their own environment and identify any barriers in the natural context [5,6]. Recently published systematic reviews concluded that telerehabilitation for adults after ABI may have equal or even better outcomes than face-to-face interventions [1,2]. However, other systematic reviews and meta-analyses concluded that the evidence available is inconclusive and insufficient [3,4]. The application of telerehabilitation has intensified with the rapid shift to telemedicine brought on by the COVID-19 pandemic, requiring healthcare professionals to adapt to caring for individuals remotely [6].

ABI, which includes stroke and traumatic brain injury (TBI), is a major health issue and a leading cause of long-term functional limitations and participation restrictions in daily life [7,8]. Therefore, ABI survivors most often require long-term care and follow-up; however, there is a lack of sufficient rehabilitation services offering continued support for this population in the chronic phase [7,9,10,11]. The advantages of telerehabilitation provide an opportunity to overcome this gap and improve accessibility to rehabilitation services later on in the rehabilitation process, thus facilitating improvement in the daily life participation of this population in the community.

**Figure 1:**
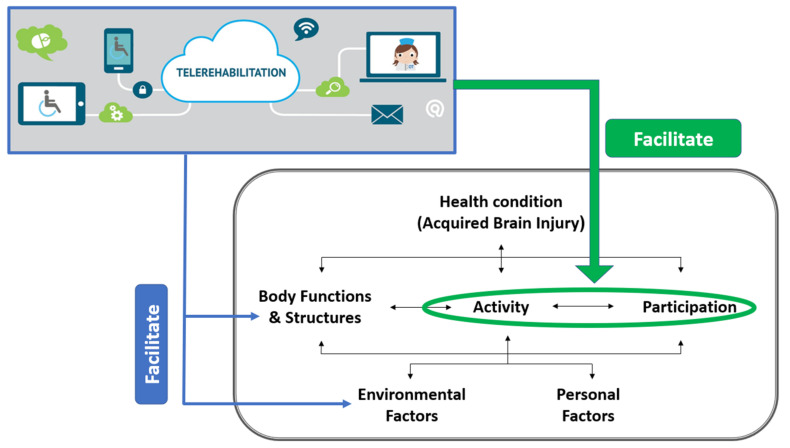
Telerehabilitation and the International Classification of Functioning, Disability, and Health (ICF): Licence: CC BY-NC-SA 3.0 IGO [12]—adapted from [26]. The green line represents the current research focus of using the advantages of telerehabilitation to directly improve activity and participation in daily life.

Participation is a core concept in the International Classification of Functioning, Disability and Health (ICF) and is broadly defined as involvement in life situations, including roles and activities [12]. It is a central concept in healthcare and is widely regarded as the ultimate goal of rehabilitation following ABI [13,14]. In line with this, the current recommendations for rehabilitation guidelines in the chronic phase after ABI include the use of long-term performance-based and client-centered interventions to facilitate participation and community reintegration [15,16,17]. Despite these recommendations, the evidence regarding interventions to enhance participation in the long-term after ABI is lacking. Many rehabilitation studies do not focus on participation outcomes but rather continue to measure and intervene at the level of body functions [13,18,19]. The same problem recurs in the telerehabilitation field; thus, most studies that examined the efficacy of telerehabilitation programs for this population focus on body functions such as balance, upper-extremity function, and cognitive skills [20,21,22,23,24,25]. Therefore, there is a need for developing telerehabilitation intervention models to facilitate participation and community reintegration for this population that extend beyond impairment remediation approaches (see Figure 1).

The Cognitive Orientation to Daily Occupational Performance (CO-OP) approach is an appropriate treatment option to meet this need. The CO-OP is a metacognitive approach that focuses on strategy training and problem solving to improve the participation in daily activities, as opposed to training directed at improving the underlying impaired body functions. It was adapted for use in different populations including adults following ABI [18,19]. Several studies have demonstrated the efficacy of using the CO-OP approach to promote personal functional goals and positively influence participation for this population. Moreover, evidence of transfer effects of CO-OP can be seen in improving performance on untrained tasks resulting from skill acquisition and learned cognitive strategies [27,28,29,30,31,32,33,34].

The use of the CO-OP remotely (Tele-CO-OP) enables the application of the intervention in an accessible manner in the home environment. The feasibility and the potential long-term benefits of the tele-CO-OP were demonstrated in a few small pilot studies among patients with various chronic health conditions [35,36,37]. Tele-CO-OP meets the definition of a complex intervention; therefore, the development and evaluation process can be based on the Medical Research Council (MRC) guidelines [38]. Following the development of the intervention, we conducted a feasibility study [39]. The tele-CO-OP was found to be feasible among adults in the chronic phase after ABI (*N* = 5) who expressed a high degree of satisfaction with both the intervention itself and the technology used in its delivery. The results showed the potential of the intervention to positively affect the participation of adults post-ABI in the community and encouraged us to continue and conduct this pragmatic exploratory pilot randomized controlled trial (RCT). In light of this, the aim of this study was to pilot the RCT to explore the clinical efficacy of the tele-CO-OP intervention for adults in the chronic phase after ABI. We will explore whether this intervention can improve the performance of daily activities and participation in community life, when compared to a waitlist control group. The results of this pilot study will be used to determine the sample size for a future larger RCT that will evaluate the effectiveness of tele-CO-OP.

## 2. Materials and Methods

### 2.1. Design and Participants

This pilot study used a pragmatic exploratory partial RCT with a waitlist control crossover design. Eight individuals were allocated to tele-CO-OP group and eight to the waitlist control group. The first six participants were not randomized (see procedure chapter).

Participants were recruited from three day-rehabilitation centers in Israel with the approval of the research ethics committees of Hadassah-Hebrew University Medical Center, Jerusalem, and Maccabi Healthcare Services, Bat-Yam, Israel (ethical committee registration numbers: 0689-15-HMO and 192016, respectively). Written informed consent was provided by all participants, and the study was conducted in accordance with the Declaration of Helsinki.

People who met the following inclusion criteria were included: (1) ≥6 months post-ABI; (2) age ≥ 18 years; (3) sufficient proficiency in Hebrew or English to participate in the study; (4) modified Rankin scale (mRS) scores of 2–4 reflecting slight to moderate disability [40]; (5) self-reported unmet functional goals; (6) internet access at home; and (7) had an adult significant other who agreed to be involved in the study. Participants were excluded with the following criteria: (1) moderate or severe aphasia; (2) a score of <21 on the Mini Mental Status Examination (MMSE); (3) a dementia diagnosis; (4) an acute illness that significantly impacts the ability to participate in the study.

### 2.2. Procedure

The study procedure began following completion of out-patient occupational therapy treatment in the rehabilitation centers. The first six participants were allocated to the tele-CO-OP or control group according to their preference, without randomization. Three of them received the teleintervention and were included in the feasibility study. Their data were included in the current study, as the intervention was identical [41]. The other three participants agreed to be included in the control group and did not want to receive the remote intervention. Their reasons for declining the teleintervention included cultural–religious reasons, fears associated with digital documentation, and a preference for face-to-face care. The next 10 participants were randomized using the minimization randomization technique of the WINPEPI software [42] to minimize differences in age and mRS levels between the groups at the baseline. This partially randomized preference design is relevant for complex intervention research [43] and technology-based intervention research, which are known for their recruitment challenges [44]. This design allowed us to collect data from participants who would otherwise not have been observed.

First, a baseline assessment was conducted, which consisted of two sessions (1.5 h each session). This assessment was administered by the occupational therapist (OT) who would be treating the participant later on, to establish a therapeutic relationship before beginning the remote intervention. Randomization was done at the end of the baseline assessment. After allocation, participants in the tele-CO-OP group received the intervention via weekly videoconference sessions over a period of three months. The intervention was administered in a telerehabilitation format using Skype ^TM^ or Zoom software.

Each participant identified five functional goals during the baseline assessment using the COPM, of which three were directly addressed during the intervention (trained goals) and two were not addressed (untrained goals), to allow assessment of generalization and transfer of learning. In the video sessions, the OT guided the participant in the use of a global problem-solving strategy (Goal-Plan-Do-Check) to help them discover their performance problems as well as potential task-specific strategies to improve their performance and enable goal attainment. The intervention protocol is described in more detail elsewhere [39]. The waitlist control group did not receive the intervention during the same time period, thus ensuring that the outcome was due to the treatment and not due to spontaneous recovery. At the end of the 3-month intervention/waitlist period, the second assessment was conducted for both groups. Due to the nature of some of the outcome measures, a blind assessment was not possible. However, the assessment was done by a different OT, who did not conduct the intervention, in order to reduce participant bias.

Following the second assessment, there was a crossover, and the control group received the same tele-CO-OP intervention for 3 months, while the tele-CO-OP group did not receive any treatment during this time. At the end of this 3-month period, the participants were assessed for the third time, by the same OT who conducted the second assessment. This assessment served as a 3-month follow-up assessment for the tele-CO-OP group and as the post-intervention assessment for the waitlist control group. All assessments were conducted in the participants’ homes with the exception of two participants who performed assessments remotely via videoconference due to the COVID-19 outbreak.

### 2.3. Measures

Sociodemographic and clinical characteristics of the participants were documented and included information such as age, education level, work status, ABI type and side, time since ABI, cognitive-screening test scores, and functional status. We collected the information from the participants using a background questionnaire created for this study and through a review of medical records. During the study neither group was prevented from utilizing additional rehabilitation services; therefore, self-report regarding use of these services was documented at post-intervention.

#### 2.3.1. Primary Outcomes

Our primary outcomes were activity performance and participation, which were measured using three instruments reflecting the perceptions of the participant, the clinician, and the significant other. Activity performance was measured with the Canadian Occupational Performance Measure (COPM) [45] and the Performance Quality Rating Scale (PQRS) [46]. The COPM is a semi-structured interview to facilitate client-centered goal setting and measure the client’s perceived occupational performance and satisfaction levels. Occupational performance problems are identified, prioritized, and rated on a 10-point rating scales (1—not able to do it/not satisfied at all; 10—able to do it extremely well/extremely satisfied). In our study, the COPM was used to formulate five goals, of which three were directly addressed during the intervention process. Changes on the other two goals were considered evidence of “far transfer” of training effects because they were not discussed or trained in the intervention. A change of ≥2 points is considered a clinically significant change. The COPM is a reliable and valid outcome measure widely used with adults after ABI [45,47,48,49].

The PQRS is an observational measure of activity performance. The OT ranks the performance level of the patient-selected, personally meaningful activities that were defined using the COPM. A 10-point rating scale is used (1—no activity criteria are met; 10—all activity criteria are met), similar to the COPM scale. The PQRS has moderate to substantial reliability and good internal responsiveness [50]. In our study, the OT rated the activity performance of the participant-chosen goals based on live observation of the performance or based on the participants’ detailed description of the performance if observation was not possible. Therefore, the OT used the PQRS only for participants who received the intervention (either before or after the crossover) and only for trained goals. The final scores reported for the COPM and PQRS are the average of scores.

Participation was measured using the Mayo-Portland Adaptability Inventory-Participation index (MPAI-4-P). The MPAI-4 measures the recovery progress of people after an ABI and includes 29 items grouped into three subscales: (a) Ability (e.g., motor, sensory, and cognitive abilities), (b) adaptation (e.g., emotional state and social interactions), and (c) participation (e.g., leisure activities, employment, and transportation). The items are rated on a scale of 0–4, with higher scores indicating more problems and limitations. The total score reflects overall disability [51,52]. The scores are converted into standardized T-scores that represent different levels of participation: below 30 = relatively good participation; 30–40 = mild limitations; 40–50 = mild to moderate limitations; 50–60 = moderate to severe limitations; above 60 = severe limitations [52]. In this study, participants completed the participation index (which includes eight items), while significant others completed the full questionnaire. The MPAI-4 is widely used by rehabilitation professionals to evaluate individuals after ABI, and there is substantial evidence that it provides satisfactory internal consistency (Cronbach’s *α* = 0.85–0.90) [53], as well as high construct, concurrent, and predictive validity for the full questionnaire and its subscales [51,54,55]. Further, the MPAI-4 shows sensitivity to clinical change after rehabilitation [56,57].

#### 2.3.2. Secondary Outcomes

Secondary outcomes included executive function in daily life, general self-efficacy, community mobility, and caregiver burden. Executive function in daily life was measured using the Dysexecutive Questionnaire (DEX) [58]. The DEX is a 20-item questionnaire to assess executive function in daily life among people with ABI. Items are rated from 0 to 4 according to the frequency with which the problem is manifested in everyday life. Higher scores indicate more problems with executive function in the real world [58]. Researchers found the DEX to have adequate concurrent [59] and ecological validity and good internal consistency (Cronbach’s *α* = 0.89) [60]. The DEX also significantly distinguished patients with brain injury of different etiologies from healthy controls [59,60,61].

General self-efficacy was measured with the New General Self-Efficacy Scale (NGSE) [62]. This is an eight-item questionnaire that assesses people’s confidence in their ability to accomplish their goals, regardless of the obstacles they may face (e.g., “Even when things are tough, I can perform quite well”). Each item is rated on a five-point scale (1 = strongly disagree; 5 = strongly agree). The score is calculated by taking the average of the ratings, with higher scores indicating higher levels of general self-efficacy. The questionnaire has excellent content validity, very good internal consistency (Cronbach’s *α* = 0.85–0.90), and sufficient test–retest reliability [62,63].

Community mobility was measured with the frequency of leaving one’s house measure [64,65]. As part of the background questionnaire, we asked how often the participants usually left the house (how many days a week). Based on the reported responses, answers were grouped as daily or nearly daily (6–7 days a week), often (2–5 days a week), and rarely (≤1 time a week). A similar definition was used in previous studies with community-dwelling older adults [64,65].

Caregiver burden was measured using the Short Form Zarit Burden Interview (ZBI-12) [66]. Primary caregivers are asked to rate 12 items on the questionnaire. The items refer to the feelings that the primary caregiver may have towards the person he or she is caring for and the effects that the primary caregiver role may have on different areas of their life. Each item is rated on a scale of 1 (never) to 5 (almost always). The final score is created from the sum of the scores of all the items, in the range of 12–60, and a higher score means a higher sense of burden. The ZBI-12 is widely used around the world, and several studies have used it in different care settings [67]. There is evidence that it has demonstrated good psychometric properties with very good internal consistency (Cronbach’s *α* = 0.85–0.89) and good convergent and exploratory validity [68]. This measure is sensitive and effective for evaluating the overall burden among caregivers of older adults, and it has good discriminative ability of caregivers of patients with dementia, advanced cancer, and acquired brain injury [69].

We also measured the participants’ satisfaction with the tele-CO-OP intervention using a satisfaction questionnaire we created for the study. This questionnaire includes 13 statements rated on a 5-point scale with higher scores reflecting greater satisfaction with the intervention [39].

### 2.4. Statistical Analysis

Analysis was conducted with SPSS Version 25.0 (IBM Corporation, Armonk, NY, USA). Descriptive statistics were used to describe the participants’ characteristics and individual COPM and PQRS scores overtime. Nonparametric statistics were used due to the small sample size, as well as the findings of non-normal distribution for some of the variables. Between-group comparisons were done with the Mann–Whitney U test for continuous data and the Fisher’s exact test for categorical data. The Mann–Whitney U test was also used to compare changes between the tele-CO-OP and the waitlist control groups on the intervention outcomes from the baseline to the post-intervention/wait period. The Wilcoxon signed-rank test was used to investigate within-group differences. Due to the small sample size, these analyses were exploratory. In addition, an effect size (*r*) was calculated from the *z* value of the Wilcoxon signed-rank test using the formula r=z/√n [70] and can be interpreted as a small (*r* ≤ |0.10|), medium (*r* = |0.30|), and large (*r* ≥ |0.50|) effect size [71]. For within- and between-group comparisons, the significance was set at *p* < 0.05.

Two of the outcome measures had missing data: the MPAI-4 and the ZBI, which were both filled out by the significant others. Missing data were dealt with in two ways. In cases where less than 20% of the items were missing, and were needed in order to compute the final score, we used the item-level person mean imputation (proration) method [72]. In cases where the complete measure score was missing at the post-intervention assessment, a scale-level imputation method was used and the last observation was carried forward [73].

## 3. Results

**Figure 2:**
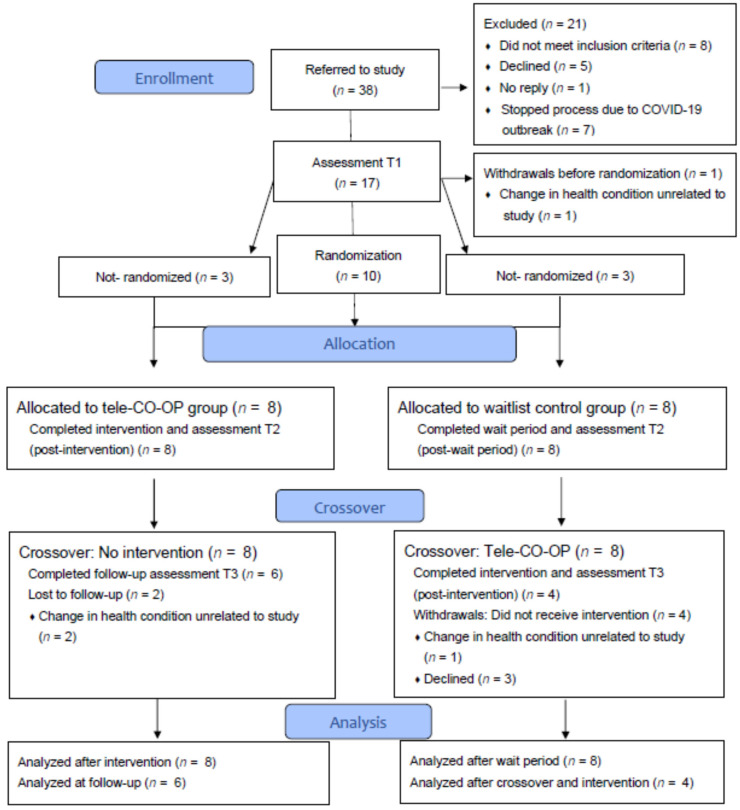
Consort flow diagram describing flow of participants through the study.

A total of 17 participants were recruited for this pilot exploratory study. Figure 2 shows the details of recruitment and the flow of participants through the study. Recruitment of participants was limited due to the outbreak of the COVID-19 pandemic, which occurred during the peak of the recruitment period. As a result of the outbreak, admission to and service accessibility at rehabilitation centers were restricted, and recruitment of seven potentially interested patients was halted. Eight participants were allocated to the intervention group and eight to the waitlist control group. One participant withdrew before group allocation, and three others withdrew after group crossover, all for health issues unrelated to the study.

Between-group comparisons at the baseline using the Mann–Whitney U test and the Fisher’s exact test revealed no significant differences in sociodemographic and clinical characteristics, including age and sex (see Table 1), except for a significant difference in the side of the ABI (*p* = 0.041).

In addition, no significant differences were found between the groups in the outcome measures. The final sample (*n* = 16) included three women (18.8%) and 13 men (81.2%), with a median age of 65.5 (IQR: 56.0–69.3) years. Fifteen participants (93.7%) were married and identified their spouse as their significant other. Twelve participants (75%) had an ischemic stroke; two participants (12.5%) had a hemorrhagic stroke; and two participants (12.5%) had a TBI. It was the first ABI for 13 of the participants (81.2%), and the median time since the ABI was 8.5 (IQR: 7.0–10.8) months. Global cognitive status was high with a median MMSE score of 28.0 (IQR: 27.3–29.0).

At the baseline, each participant identified five goals related to improving their functioning in everyday life. Overall, the goals covered all nine activity and participation domains according to the ICF [12], such as self-care (e.g., dry my back independently after taking a shower and dress independently), domestic life (e.g., cook a simple dish on my own and help prepare the Sabbath meal), mobility (e.g., resume riding my bicycle every day and resume driving), communication (e.g., type with fewer mistakes and stay concentrated in a conversation), major life areas (e.g., start volunteering and get to work on time), and community, social, and civic life (e.g., go to the mall with a friend and start going to an art class).

The Mann–Whitney U test was used to investigate between-group change scores’ differences. The results suggested that participants in the tele-CO–OP group showed greater improvement in COPM performance and satisfaction scores and in the MPAI-4-P scores compared with the waitlist control group participants; however, these improvements were not statistically significant. It is notable that the difference in COPM performance improvement between the groups was on the threshold of statistical significance (*U* = 13.5, *p* = 0.051).

In addition, within-group analyses were performed post-intervention/wait-period using the Wilcoxon signed-rank test for each group separately (see Table 2). No significant improvements were found in the control group at the end of the 3-month wait period, whereas the tele-CO-OP group showed statistically significant improvements in COPM performance (*z* = −2.52, *p* = 0.012) and satisfaction (*z* = −2.20, *p* = 0.028) ratings. It should be noted that during the first three months, there were participants in both groups who received other community occupational therapy (one participant in the study group and two in the control group) or physiotherapy services (six participants in the study group and four in the control group). According to the Fisher’s exact test, the number of participants who received additional rehabilitation services did not differ significantly between the groups. Therefore, to maximize power, we pooled the data of the intervention group participants (*n* = 8) with the control group participants who received the intervention after the crossover point (*n* = 4). Thus, analyses to explore potential intervention effectiveness were conducted for this combined tele-CO-OP group (*n* = 12).

For the combined tele-CO-OP group, the median number of CO-OP sessions per participant was 10.50 (IQR: 9.25–13.00). The median time for the complete CO-OP teleintervention was 8.04 h (IQR: 6.35–11.58). No safety concerns or adverse events were related to study participation. Wilcoxon signed-rank tests revealed statistically significant within-group changes from the baseline to post-intervention for all primary outcome measures (Table 3). COPM participant ratings showed significant improvements with large effect sizes for both trained and untrained goals. The PQRS OT ratings showed similar results for the trained goals.

The MPAI-4-P rated by participants showed a significant improvement and a moderate effect size. The decrease in the median MPAI-4-P score from 45 to 39 reflects a reduction in the level of participation limitation based on the score ranges described in the manual [52]. Similar results were found for the total MPAI-4 score rated by the significant other.

**Figure 3:**
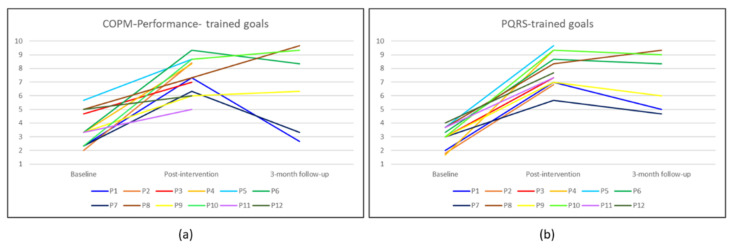
Mean scores over time for trained goals per participant (P): (**a**) Mean COPM-Performance scores for trained goals rated by the participants; (**b**) mean PQRS scores for trained goals rated by the occupational therapist.

In terms of a clinically significant change, the improvement in the average COPM and PQRS scores were of at least three points and in some cases even four points, which are considered clinically meaningful (more than two points). In addition, individual profiles showed the functional performance gains of the trained goals over time, as seen in Figure 3. 

Overall, participants’ individual profiles corroborated the statistical trends. As can be seen in Figure 3a, the mean COPM-Performance scores indicated clinically significant improvement (≥2 points) at the post-intervention assessment in ten of the 12 participants (except participants 11 and 12). Four of six participants maintained or improved their scores at the 3-month follow-up. Figure 3b illustrates that individual gains for the same trained goals of each participant were even more evident in the OT mean PQRS rating. As shown in the figure, all 12 participants had clinically significant improvements in PQRS scores after the intervention. At the 3-month follow-up, the results were similar to the COPM-Performance results, indicating partial maintenance of the improvements.

As for the secondary outcomes, the results indicated that the frequency of leaving the house had improved significantly post-intervention; ten participants (83.3%) reported leaving the house 6–7 days a week post-intervention, compared with six participants (50%) at baseline. We did not find significant changes in the other secondary outcomes; however, there was a trend of improvement in general self-efficacy ratings on the NGSE, with a moderate effect size.

The improvements were partially maintained at the follow-up (Table 3). After the crossover, two participants from the intervention group were lost to the follow-up, and four participants from the control group did not receive the intervention after the wait period (Figure 2); thus, the follow-up group included six participants. Despite the small sample, we found that the significant improvements in the COPM and PQRS scores were maintained at the follow-up. In addition, we found a trend towards significance for MPAI-4 scores.

All participants completed the intervention satisfaction questionnaire at the end of the intervention period. Mean ratings for each item can be seen in Table 4. Overall, high to very high satisfaction was reported for 11/13 statements representing various aspects of the treatment. Items with the highest scores (≥4.5) included items of general satisfaction (item 1); enjoyment (item 2); satisfaction with the course of treatment (item 3); satisfaction with the treatment approach (item 4); satisfaction with the level of involvement of the significant other (item 7); desire to use this service again if it were possible (item 11); and likeliness of recommending the treatment program to a person with a similar health condition (item 12).

Finally, we used the findings from this pilot study to calculate the sample size for a full future RCT. We based our sample-size estimate on the most conservative effect size found in our primary outcome results (MPAI-4-P; *ES*= 0.43) with a two-sided significance level of 0.05 and power of 80% with equal allocation to two arms, which would require 23 patients in each group. Considering a drop-out rate of 25%, as found in the current study, 58 participants should be recruited in total (29 per arm).

## 4. Discussion

This pragmatic exploratory pilot study set out to examine the initial efficacy of the tele-CO-OP intervention on activity performance and participation among adults in the chronic phase after ABI compared to a waitlist control group. The results indicated there were no statistically significant between-group differences in the outcome-measure-change scores at the end of the intervention/wait period. It is possible that this is due to the small sample size. The results of within-group analysis indicated some significant improvements in activity performance in the tele-CO-OP group post-intervention; however, no significant improvements were found in the control group after a 3-month wait period. After pooling the data of all the participants who completed the intervention to a combined tele-CO-OP group, we found statically significant improvements in all the primary outcome measures indicating improvements in activity performance on both trained and untrained goals. Significant improvements were found in participation as well. In addition, the results showed significant improvements in the frequency of leaving the house post-intervention, with no other significant improvements in the secondary outcomes. The results were partially maintained at the 3-month follow-up. Our pilot RCT showed satisfactory retention and adherence rates, and no adverse events related to the intervention were reported during the study. In addition, participants reported a high degree of satisfaction with the remote intervention. Based on the results of this pilot study, we calculated the sample size for a larger RCT to assess the effectiveness of tele-CO-OP in the future prior to the next step of clinical implementation.

The current pilot study adds to the body of literature demonstrating that the CO-OP intervention is feasible and effective in improving daily function in selected individual goals for adults in the chronic phase after ABI. The group results, as well as the individual results, are in line with previous traditional face-to-face CO-OP studies with this population [27,29,31,33,34,74,75,76]. In addition, our preliminary results corroborated previous CO-OP telerehabilitation feasibility studies that reported improved daily function among adults in the chronic phase after ABI [32,39] and individuals with other health conditions, including cancer survivors with cognitive decline [36], older adults with subjective cognitive complaints [35], and adolescents with myelomeningocele [37]. Moreover, this is the first pilot study to evaluate the benefits of tele-CO-OP in comparison to a control group, thus extending the previous quasi-experimental tele-CO-OP findings. Considering that the control group did not show improvement in outcome measures at the end of the waiting period, this may imply that the improvement in the tele-CO-OP group is due to the intervention and not to spontaneous recovery or other alternative factors, such as interventions received in the community. It is noteworthy that the COPM self-ratings of satisfaction with performance in the control group were on the threshold of being significantly improved (*p* = 0.05). A possible explanation is that the very act of goal setting resulted in people attending and promoting these goals. There is literature that suggests that goal setting in itself can be a therapeutic process [77,78].

The initial results of this exploratory pilot study indicated significant improvements in the trained goals and also in the untrained goals, suggesting that the strategies the participants learned and applied during the intervention may have been used in daily activities that were not directly addressed. In particular, explicit metacognitive strategies applied in the CO-OP approach seem promising for enhancing transfer and generalization to completely unrelated tasks. This can be expected because the therapist is more concerned with teaching problem-solving skills than with teaching the specific functional skill [79,80]. In addition, generalization and transfer of skills can potentially be enhanced when activities are relevant to the individual and when therapy is provided in the individual’s own environment [80], which was possible in this study due to the remote implementation of the intervention. Additional evidence of possible transfer was also found in previous studies [27,29,32,33,39]. Further support for the transfer process was evident in a qualitative study with five adults in the chronic phase following ABI who explicitly reported learning and transferring the strategies they were taught in the CO-OP intervention to other daily activities [81]. We heard similar reports from some of the participants in the current study.

Our results also indicated significant improvements in participation following the intervention, as reported by the participants and their significant others. Participation, as measured by the MPAI-4, is a general outcome that encompass a wide range of life domains. Therefore, it may be more difficult to affect because performance improvements in specific goals may not necessarily translate into global outcomes like participation [39,82,83]. These results may also be explained by the transfer and generalization process that can result in broader improvements as reflected in participation measures. Our results are in line with previous studies that found positive effects of the CO-OP in community-participation measures [32,33,76].

Two unique aspects of this pilot study design further validate our results. First, this study evaluated the potential efficacy of the intervention from three points of view: the participant, the significant other, and the clinician. All three sources indicated a positive impact of the intervention on activity performance and participation. This adds to the current face-to-face CO-OP studies that use one or two perspectives, usually the participant and the clinician [27,31,74,75,84] or the significant other [29,32,34]. Second, we conducted a three-month follow-up assessment for six participants, which indicated partial maintenance of the results. While gains achieved in the individual goals were maintained, the improvements in general participation and the secondary outcomes were not. The results of this pilot study add to the limited evidence from pilot studies of the CO-OP intervention suggesting some maintenance of training benefits over time [29,32,34].

Another interesting finding of our study was that the frequency of leaving the house increased significantly after the tele-CO-OP intervention. Frequency of leaving the house is a simple pragmatic measure whose improvement may be significant because of its association with function, health status, and mortality among community-residing older adults [64,65,85]. Leaving one’s home is important and provides opportunities to engage in a variety of activities that involve social, cognitive, productive, emotional, and other aspects [64]. In the current pilot study, some of the participants expressed dissatisfaction with the fact that they spend most days at home and expressed a desire to get out of the house more often. Each participant in the combined tele-CO-OP group had at least one goal that involved leaving the house either directly (e.g., “to go out on my own more” and “to find a productive activity out of the house 2–3 times a week”) or indirectly (e.g., “to start volunteering once a week” and “to go back to the community center twice a week”); overall, 29 goals (50%) involved leaving the house. Thus, the improvement we found in participants’ performance on their selected goals may have contributed to the increase in the frequency of leaving the house in this sample. Despite this being a simple outcome measure, it may have meaningful implications for adults dealing with chronic consequences of ABI, and there is a need for more research regarding this measure as a rehabilitation outcome in this population. We would point out that this issue is even more urgent now that the COVID-19 pandemic is causing an increase in homebound older adults [86,87]. During the COVID era, social isolation has become the preferred default option, particularly for people with recent morbidity, such as the subjects included in our study. Moreover, due to social distancing and the reduced availability of community-based services, telehealth innovations are crucial for closing this gap for this vulnerable population [85].

The CO-OP approach uses guided discovery, whereby the therapist guides the participant in identifying the strategies that can solve their performance problem. It is postulated that in this way, the participant can attribute the improvements in function to their actions in the intervention rather than to an external factor, thereby increasing self-efficacy [28,29,88]. Therefore, in this pilot study we measured general self-efficacy; yet, we did not find a significant improvement following the intervention. There is some evidence of improved self-efficacy after CO-OP interventions with adults in the subacute [28] and chronic phase after stroke [29]; however, they used measures of self-efficacy in daily activities and not general self-efficacy as in our study. In a recent CO-OP study with children referred for motor-coordination difficulties, self-efficacy was measured specifically for each goal [89]. They found significant improvements post intervention in the directly addressed goals but not in the untrained goals. This might suggest that self-efficacy improves in activities that are addressed in the intervention but does not necessarily extend to other activities. That may explain why the increase in general self-efficacy in the current study was not significant.

No statistically significant within-group differences were found on the other two secondary outcomes of executive function in daily activities (DEX) and caregiver burden (ZBI-12). Some earlier studies with a similar population after ABI did not find statistically significant improvements in the DEX after CO-OP treatment [29,33]. These results can be explained by the small sample size or the relatively high scores on these measures at baseline, which may indicate fewer executive-function problems in daily life and a smaller caregiver burden. This may be due to the current study’s sample characteristics that included participants that were independent or needed mild assistance in basic activities of daily living.

The results in this exploratory pilot study should be interpreted cautiously due to some limitations. First, a major limitation of the study was the partial randomization design, which might mask inherent differences between the groups. Our efforts to address this limitation led to the use of the minimization randomization technique to minimize differences in age and disability levels between the groups. In addition, the small sample size of this study limits the power and the generalizability of the study results, warranting further large-scaled studies. Furthermore, the absence of an active treatment control group limits our ability to identify the active ingredients in the intervention beyond the clinician’s attention. Additionally, despite the fact that all the goals addressed daily function, we did not compare the complexity and difficulty of the goals between the groups (e.g., “drying my back after the shower” vs. “starting to volunteer”). Additionally, the second and third assessments were conducted by a second OT, who did not conduct the intervention and who was not blinded to group allocation, introducing a potential scoring bias; yet, most assessments were self-report or based on open questions, therefore reducing the problematic nature of this matter. Another limitation is that the PQRS, which is usually rated based on observation, was rated in this study based on observation of the goal performance or based on a description of the performance by the participants if observation was not possible (e.g., in cases where the activity is not performed at home). Despite these limitations, the preliminary study results are intriguing, and they indicate that a larger, more-comprehensive trial should be conducted. We encountered quite a few challenges implementing a rigorous RCT, which resulted in some of the study limitations outlined here. In future studies, it may be worthwhile to explore alternative approaches to generating research evidence for technology-based interventions in the rehabilitation field [44].

## 5. Conclusions

This pragmatic exploratory pilot study demonstrated the feasibility and preliminary efficacy of an activity-based teleintervention in promoting daily function and participation among adults in the chronic phase after ABI. We can use the results of this pilot study to develop a larger RCT in the future to evaluate the effectiveness of the tele-CO-OP as a basis for its implementation in clinical settings [38]. Participants who completed the intervention program reported high levels of satisfaction with the remote treatment. On the other hand, some participants were not interested in receiving remote services. However, it should be noted that recruitment for the study was prior to the COVID-19 outbreak; therefore, following the changes brought about by the pandemic, it may be possible that patients will be more open to receiving remote rehabilitation today. Regardless, as with other interventions, this tele-CO-OP is not suitable for everyone, so its use should be guided by clinical reasoning. Furthermore, we hope that our findings and results from additional telerehabilitation studies will further support the integration of remote rehabilitation services into standard clinical care for adults after ABI living in the community.

## Figures and Tables

**Table 1:** Sociodemographic and clinical characteristics at baseline for tele-CO-OP and waitlist control groups.

	Tele-CO-OP (*n* = 8) Median (IQR) or *n* (%)	Waitlist Control (*n* = 8) Median (IQR) or *n* (%)	*p*
**Sociodemographic Characteristics**			
Age	64.0 (51.8–67.0)	65.5 (63.5–70.0)	0.494
Sex Female Male	1 (12.5%) 7 (87.5%)	2 (25.0%) 6 (75.0%)	1.000
Education (years)	12.5 (10.3–14.5)	12.5 (11.3–15.5)	0.525
Marital status Married Widowed	8 (100.0%)	7 (87.5%) 1 (12.5%)	1.000
Significant other Spouse Child	8 (100.0%)	7 (87.5%) 1 (12.5%)	1.000
Living status With spouse and/or children Alone	8 (100.0%)	7 (87.5%) 1 (12.5%)	1.000
Living area Urban Rural	7 (87.5%) 1 (12.5%)	8 (100.0%)	1.000
Work status Working Not working	(25.0%) 6 (75.0%)	2 (25.0%) 6 (75.0%)	1.000
**Clinical Characteristics**			
Type of ABI Ischemic stroke Hemorrhagic stroke Traumatic brain injury	6 (75.0%) 2 (25.0%)	6 (75.0%) 2 (25.0%)	0.467
Side of ABI Right Left Bilateral	2 (25.0%) 5 (62.5%) 1 (12.5%)	7 (87.5%) 1 (12.5%)	0.041
First ABI Yes No	6 (75.0%) 2 (25.0%)	7 (87.5%) 1 (12.5%)	1.000
Time since ABI (months)	8.0 (7.0–9.8)	9.5 (6.3–11.8)	0.560
Outpatient rehabilitation period (months)	4.5 (2.3–6.0)	4.0 (2.0–5.8)	0.707
MMSE	28.0 (25.5–29.0)	28.5 (28.0–30.0)	0.161
Modified Rankin Scale (mRS)	3.0 (2.0–3.0)	3.0 (2.3–3.8)	0.492
Walking aid Yes No	4 (50.0%) 4 (50.0%)	4 (50.0%) 4 (50.0%)	1.000
Community mobility by driving car post-ABI Yes No	4 (50.0%) 4 (50.0%)	2 (25.0%) 6 (75.0%)	0.608
Community mobility by foot post-ABI Yes No	8 (100.0%) -	6 (75.0%) 2 (25.0%)	0.467
Previous computer use experience Yes No	7 (87.5%) 1 (12.5%)	6 (75.0%) 2 (25.0%)	1.000
Previous video-conference App use experience Yes No	4 (50.0%) 4 (50.0%)	3 (37.5%) 5 (62.5%)	1.000

Note. Statistical tests used were Fisher’s Exact Test or Mann–Whitney U test as appropriate. IQR = interquartile range; ABI = acquired brain injury; MMSE = mini mental state examination.

**Table 2:** Within-group T1 (pre)–T2 (post) differences in outcome measures for tele-CO-OP and waitlist control groups.

Outcome	Tele-CO-OP (*n* = 8)			Waitlist Control (*n* = 8)		
	T1 Median (IQR)	T2 Median (IQR)	*p*	T1 Median (IQR)	T2 Median (IQR)	*p*
COPM(P) COPM(S)	2.8 (2.3–3.3) 2.5 (2.3–5.1)	7.3 (6.1–8.6) 6.8 (5.5–9.3)	0.012 0.028	3.8 (2.3–5.5) 4.0 (2.8–5.3)	5.2 (4.1–7.8) 6.2 (3.3–7.3)	0.106 0.050
MPAI-4-P	45.5 (41.0–52.0)	42.5 (30.0–49.8)	0.123	47.0 (43.5–48.0)	45.0 (41.0–50.5)	0.553
MPAI-4-SO-total score	45.0 (41.3–53.0)	45.0 (38.0–51.3)	0.074	42.0 (39.3–45.8)	42.5 (37.5–48.5)	0.933
DEX	16.5 (4.8–28.8)	17.0 (7.5–22.8)	0.482	10.5 (1.8–20.8)	8.0 (3.0–12.3)	0.932
NGSE	3.7 (3.6–4.1)	4.2 (2.9–4.5)	0.307	3.6 (3.0–3.8)	3.3 (3.0–3.6)	0.396
ZBI	23.0 (17.5–31.8)	21.5 (16.0–34.0)	0.916	20.0 (18.0–25.0)	18.0 (12.8–19.5)	0.075
Frequency of leaving the house Daily or nearly daily Often Rarely	5 (62.5%) 2 (25.0%) 1 (12.5%)	6 (75.0%) 1 (12.5%) 1 (12.5%)	0.317	3 (37.5%) 5 (62.5%)	3 (37.5%) 5 (62.5%)	1.000

Note. Wilcoxon signed-rank tests were performed for within-group analysis, and medians and interquartile ranges (IQR) are presented. COPM = Canadian Occupational Performance Measure; these scores are the mean of the three first COPM goals that were trained in the tele-CO-OP group; (P) performance score; (S) = satisfaction score; MPAI-4-P = Mayo-Portland Adaptability Inventory-4-Participation Index; SO = significant other; DEX = Dysexecutive Questionnaire; NGSE = New General Self-Efficacy Scale; ZBI-12 = Short Form Zarit Burden Interview; frequency of leaving the house: daily or nearly daily = 6–7 days a week; often = 2–5 days a week; rarely = ≤1 day a week.

**Table 3:** Changes in outcome measures for combined tele-CO-OP group from T1 (pre-) to T2 (post-) intervention (*n* = 12) and from pre-intervention to T3 (three-month follow-up) (*n* = 6).

Outcome	T1 (*n* = 12)	T2 (*n* = 12)	T1−T2		T3 (*n* = 6)	T1−T3	
	Median (IQR) or *N* (%)	Median (IQR) or *N* (%)	*p*	*r* (*ES*)⁑	Median (IQR) or *N* (%)	*p*	*r* (*ES*)⁑
COPM(P)-Trained COPM(P)-Untrained COPM(S)-Trained COPM(S)-Untrained	3.33 (2.33–4.92) 3.00 (2.38–5.63) ^¥^ 3.47 (2.33–5.83) 2.75 (1.38–6.13) ^¥^	7.33 (6.08–8.60) 6.00 (3.88–7.88) ^¥^ 8.17 (6.08–9.58) 7.00 (5.75–8.13) ^¥^	0.002 0.012 0.005 0.012	−0.63 −0.56 −0.57 −0.56	7.33 (3.17–9.42) 6.75 (4.00–8.25) 6.67 (4.42–9.50) 7.25 (5.75–10.00)	0.028 0.043 0.046 0.028	−0.64 −0.58 −0.58 −0.64
PQRS-Trained	3.00 (2.25–3.67)	7.50 (7.00–9.17)	0.002	−0.63	7.17 (4.92–9.08) ^†^	0.027	−0.64
MPAI-4-P	45.00 (41.00–49.75)	39.00 (28.00–46.00)	0.037	−0.43	34.50 (26.00–54.25)	0.068	−0.53
MPAI-4-SO-total score	45.00 (41.00–49.75)	43.00 (35.50–48.75)	0.035	−0.43	37.00 (36.50–47.50) ^ǐ^	0.068	−0.58
DEX	11.50 (2.00–23.50)	12.50 (3.25–21.00)	0.969	−0.01	13.00 (7.50–23.25)	0.465	−0.21
NGSE	3.63 (3.19–3.97)	4.15 (3.50–4.47)	0.074	−0.37	3.63 (2.94–4.22)	0.598	−0.15
ZBI	20.00 (17.25–29.50)	18.00 (16.00–25.00)	0.573	−0.12	19.00 (15.50–28.50) ^ǐ^	0.068	−0.58
Frequency of leaving the house Daily or nearly daily Often Rarely	6 (50.0%) 5 (41.7%) 1 (8.3%)	10 (83.3%) 1 (8.3%) 1 (8.3%)	0.046		4 (66.7%) 2 (33.3%)	0.317	

Note. Wilcoxon signed-rank test was performed for within-group analysis, and medians and interquartile ranges (IQR) are presented. EF = effect size. ⁑ An effect size (*r*) was calculated from the *z* value of Wilcoxon signed-rank test (*r* = *z*/√*n*) (Fritz et al., 2012) and can be interpreted as a small (*r* ≤ 0.10), medium (*r* = 0.30), and large (*r* ≥ 0.50) effect size (Cohen, 1992). COPM = Canadian Occupational Performance Measure; (P) performance score; (S) = satisfaction score; PQRS = Performance Quality Rating Scale; MPAI-4-P = Mayo-Portland Adaptability Inventory-4-Participation Index; SO = significant other; DEX = Dysexecutive Questionnaire; NGSE = New General Self-Efficacy Scale; ZBI-12 = Short Form Zarit Burden Interview; Frequency of leaving the house: Daily or nearly daily = 6–7 days a week; often = 2–5 days a week; rarely = ≤1 day a week; ^¥^ *n* = 10; ^†^ *n* = 6; ^ǐ^ *n* = 5.

**Table 4:** Ratings of the tele-CO-OP satisfaction questionnaire items (*n* = 12).

Item		Mean ± SD
1.	In general, how satisfied are you with the treatment program you have received?	4.67 ± 0.65
2.	How much did you enjoy participating in the treatment program?	4.50 ± 0.67
3.	How satisfied are you with the course of treatment (number and length of sessions, frequency of sessions)?	4.58 ± 0.52
4.	How satisfied are you with the treatment approach used in the sessions (e.g., “Goal, Plan, Do, Check”)?	4.75 ± 0.45
5.	To what extent do you think you will continue to use the method you have learned to deal with other situations in your life?	3.92 ± 1.38
6.	How satisfied are you with having a significant other (family member/formal caregiver/other) involved in the treatment program?	4.00 ± 1.41
7.	How satisfied are you with the level of involvement of your significant other (family member/formal caregiver/other) in the treatment program?	4.55 ± 0.69
8.	How satisfied are you with the therapeutic relationship between you and the occupational therapist during the treatment program?	4.33 ± 0.89
9.	How satisfied are you with the remote treatment experience using video sessions?	4.08 ± 1.17
10.	How satisfied are you with the experience of using Zoom\Skype ^TM^ in the treatment program (in terms of ease of use, quality of image and sound)?	4.08 ± 1.17
11.	To what extent would you like to use this service again, if there were such a possibility?	4.50 ± 0.67
12.	How likely are you to recommend our treatment program to a person with a similar health condition?	4.58 ± 0.52
13.	To what extent would you prefer that the treatment had been done face-to-face?	3.92 ± 1.24

1 = very low; 2 = low; 3 = medium; 4 = high; 5 = very high.

## Data Availability

The data presented in this study are available on reasonable request from the corresponding author.

## References

[1] Knepley K.D., Mao J.Z., Wieczorek P., Okoye F.O., Jain A.P., Harel N.J. (2021). Impact of telerehabilitation for stroke-related deficits. Telemed. E-Health.

[2] Sarfo F.S., Ulasavets U., Opare-Sem O.K., Ovbiagele B. (2018). Tele-rehabilitation after stroke: An updated systematic review of the literature. J. Stroke Cerebrovasc. Dis..

[3] Appleby E., Gill S.T., Hayes L.K., Walker T.L., Walsh M., Kumar S. (2019). Effectiveness of telerehabilitation in the management of adults with stroke: A systematic review. PLoS ONE.

[4] Ownsworth T., Arnautovska U., Beadle E., Shum D.H., Moyle W. (2018). Efficacy of telerehabilitation for adults with traumatic brain injury: A systematic review. J. Head Trauma Rehabil..

[5] Cason J., Hartmann K., Jacobs K., Richmond T. (2018). Telehealth in Occupational Therapy. Am. J. Occup. Ther..

[6] Subbarao B.S., Stokke J., Martin S.J. (2021). Telerehabilitation in acquired brain injury. Phys. Med. Rehabil. Clin..

[7] Andelic N., Howe E.I., Hellstrøm T., Sanchez M.F., Lu J., Løvstad M., Røe C. (2018). Disability and quality of life 20 years after traumatic brain injury. Brain Behav..

[8] De Graaf J., Schepers V., Nijsse B., Nijsse C., Post M.W., Visser-Meily J. (2020). The influence of psychological factors and mood on the course of participation up to four years after stroke. Disabil. Rehabil..

[9] Wheeler S., Golisz K.M., Radomski M.V. (2015). Community recovery and participation. Traumatic Brain Injury (TBI): Interventions to Support Occupational Performance.

[10] Wolf T.J., Baum C.M., Lee D., Hammel J. (2016). The development of the Improving Participation after Stroke Self-Management Program (IPASS): An exploratory randomized clinical study. Top. Stroke Rehabil..

[11] Stiekema A.P., Winkens I., Ponds R., De Vugt M.E., Van Heugten C.M. (2020). Finding a new balance in life: A qualitative study on perceived long-term needs of people with acquired brain injury and partners. Brain Inj..

[12] World Health Organization (WHO) (2001). International Classification of Functioning, Disability and Health (ICF).

[13] Engel-Yeger B., Tse T., Josman N., Baum C., Carey L.M. (2018). Scoping review: The trajectory of recovery of participation outcomes following stroke. Behav. Neurol..

[14] Klepo I., Sangster Jokić C., Tršinski D. (2020). The role of occupational participation for people with traumatic brain injury: A systematic review of the literature. Disabil. Rehabil..

[15] Rodríguez-Bailón M., López-González L., Merchán-Baeza J.A. (2020). Client-centred practice in occupational therapy after stroke: A systematic review. Scand. J. Occup. Ther..

[16] Wolf T.J., Chuh A., Floyd T., McInnis K., Williams E. (2015). Effectiveness of occupation-based interventions to improve areas of occupation and social participation after stroke: An evidence-based review. Am. J. Occup. Ther..

[17] Hebert D., Lindsay M.P., McIntyre A., Kirton A., Rumney P.G., Bagg S., Bayley M., Dowlatshahi D., Dukelow S., Garnhum M. (2016). Canadian stroke best practice recommendations: Stroke rehabilitation practice guidelines, Update 2015. Int. J. Stroke.

[18] McEwen S.E., Skidmore E.R., Wolf T.J., Dawson D.R., McEwen S.E., Polatajko H.J. (2017). Using the CO-OP Approach: Stroke. Cognitive Orientation to Daily Occupational Performance in Occupational Therapy: Using the CO-OP ApproachTM to Enable Participation Across the Life Span.

[19] Dawson D.R., Hunt A.W., Polatajko H.J., Dawson D.R., McEwen S.E., Polatajko H.J. (2017). Using the CO-OP Approach: Traumatic Brain Injury. Cognitive Orientation to Daily Occupational Performance in Occupational Therapy: Using the CO-OP ApproachTM to Enable Participation Across the Life Span.

[20] Chen J., Jin W., Dong W.S., Jin Y., Qiao F.L., Zhou Y.F., Ren C.C. (2017). Effects of home-based telesupervising rehabilitation on physical function for stroke survivors with hemiplegia: A randomized controlled trial. Am. J. Phys. Med. Rehabil..

[21] Kizony R., Weiss P.L., Harel S., Feldman Y., Obuhov A., Zeilig G., Shani M. (2017). Tele-rehabilitation service delivery journey from prototype to robust in-home use. Disabil. Rehabil..

[22] Keidel M., Vauth F., Richter J., Hoffmann B., Soda H., Griewing B., Scibor M. (2017). Home-based telerehabilitation after stroke. Der Nervenarzt.

[23] Lloréns R., Noé E., Colomer C., Alcañiz M. (2015). Effectiveness, usability, and cost-benefit of a virtual reality–based telerehabilitation program for balance recovery after stroke: A randomized controlled trial. Arch. Phys. Med. Rehabil..

[24] Woolf C., Caute A., Haigh Z., Galliers J., Wilson S., Kessie A., Hirani S., Hegarty B., Marshall J. (2016). A comparison of remote therapy, face to face therapy and an attention control intervention for people with aphasia: A quasi-randomised controlled feasibility study. Clin. Rehabil..

[25] Burgos P.I., Lara O., Lavado A., Rojas-Sepúlveda I., Delgado C., Bravo E., Kamisato C., Torres J., Castañeda V., Cerda M. (2020). Exergames and telerehabilitation on smartphones to improve balance in stroke patients. Brain Sci..

[26] Almojaibel A. (2017). Understanding Intention to Use Telerehabilitation: Applicability of the Technology Acceptance Model (TAM). Ph.D. Thesis.

[27] McEwen S., Polatajko H., Huijbregts M., Ryan J. (2010). Inter-task transfer of meaningful, functional skills following a cognitive-based treatment: Results of three multiple baseline design experiments in adults with chronic stroke. Neuropsychol. Rehabil..

[28] McEwen S., Polatajko H., Baum C., Rios J., Cirone D., Doherty M., Wolf T. (2015). Combined cognitive-strategy and task-specific training improve transfer to untrained activities in subacute stroke: An exploratory randomized controlled trial. Neurorehabilit. Neural Repair.

[29] Poulin V., Korner-Bitensky N., Bherer L., Lussier M., Dawson D.R. (2017). Comparison of two cognitive interventions for adults experiencing executive dysfunction post-stroke: A pilot study. Disabil. Rehabil..

[30] Saeidi Borujeni M., Hosseini S.A., Akbarfahimi N., Ebrahimi E. (2019). Cognitive orientation to daily occupational performance approach in adults with neurological conditions: A scoping review. Med. J. Islamic Repub. Iran (MJIRI).

[31] Ahn S.-N., Yoo E.-Y., Jung M.-Y., Park H.-Y., Lee J.-Y., Choi Y.-I. (2017). Comparison of Cognitive Orientation to daily Occupational Performance and conventional occupational therapy on occupational performance in individuals with stroke: A randomized controlled trial. NeuroRehabilitation.

[32] Ng E., Polatajko H., Marziali E., Hunt A., Dawson D. (2013). Telerehabilitation for addressing executive dysfunction after traumatic brain injury. Brain Inj..

[33] Dawson D.R., Binns M.A., Hunt A., Lemsky C., Polatajko H.J. (2013). Occupation-based strategy training for adults with traumatic brain injury: A pilot study. Arch. Phys. Med. Rehabil..

[34] Dawson D.R., Gaya A., Hunt A., Levine B., Lemsky C., Polatajko H.J. (2009). Using the cognitive orientation to occupational performance (CO-OP) with adults with executive dysfunction following traumatic brain injury. Can. J. Occup. Ther..

[35] Nadler E., Linkewich B., Ng E.M., Skidmore E.R., Hunt A.W., Dawson D.R., Dawson D.R., McEwen S.E., Polatajko H.J. (2017). Using the CO-OP Approach: Alternative Delivery Methods, in Cognitive Orientation to Daily Occupational Performance in Occupational Therapy: Using the CO-OP ApproachTM to Enable Participation Across the Life Span.

[36] Maeir T., Nahum M., Makranz C., Hoba A., Peretz T., Nagary S.N., Silberman N., Gilboa Y. (2021). The feasibility of a combined model of online interventions for adults with cancer-related cognitive impairment. Br. J. Occup. Ther..

[37] Steinhart S., Raz-Silbiger S., Beeri M., Gilboa Y. (2021). Occupation based telerehabilitation intervention for adolescents with myelomeningocele: A pilot study. Phys. Occup. Ther. Pediatrics.

[38] Skivington K., Matthews L., Simpson S.A., Craig P., Baird J., Blazeby J.M., Boyd K.A., Craig N., French D.P., McIntosh E. (2021). A new framework for developing and evaluating complex interventions: Update of Medical Research Council guidance. BMJ.

[39] Beit Yosef A., Jacobs J.M., Shenkar S., Shames J., Schwartz I., Doryon Y., Khalailh F., Berrous S., Gilboa Y. (2019). Activity performance, participation, and quality of life among adults in the chronic stage after acquired brain injury-The feasibility of an occupation-based telerehabilitation Intervention. Front. Neurol..

[40] Patel N., Rao V.A., Heilman-Espinoza E.R., Lai R., Quesada R.A., Flint A.C. (2012). Simple and reliable determination of the modified rankin scale score in neurosurgical and neurological patients: The mRS-9Q. Neurosurgery.

[41] Charlesworth G., Burnell K., Hoe J., Orrell M., Russell I. (2013). Acceptance checklist for clinical effectiveness pilot trials: A systematic approach. BMC Med. Res. Methodol..

[42] Abramson J.H. (2011). WINPEPI updated: Computer programs for epidemiologists, and their teaching potential. Epidemiol. Perspect. Innov..

[43] Loeb K.L., Weissman R.S., Marcus S., Pattanayak G., Hail L., Kung K.C., Schron D., Zucker N., Le Grange D., Lock J. (2020). Family-Based Treatment for Anorexia Nervosa Symptoms in High-Risk Youth: A Partially-Randomized Preference-Design Study. Front. Psychiatry.

[44] Wang R.H., Kenyon L.K., McGilton K.S., Miller W.C., Hovanec N., Boger J., Viswanathan P., Robillard J.M., Czarnuch S.M. (2021). The time is now: A FASTER approach to generate research evidence for technology-based interventions in the field of disability and rehabilitation. Arch. Phys. Med. Rehabil..

[45] Law M.C., Baptiste S., Carswell A., McColl M.A., Polatajko H., Pollock N. (2014). Canadian Occupational Performance Measure: COPM.

[46] Miller L., Polatajko H., Missiuna C., Mandich A., Macnab J. (2001). A pilot trial of a cognitive treatment for children with developmental coordination disorder. Hum. Mov. Sci..

[47] Phipps S., Richardson P. (2007). Occupational therapy outcomes for clients with traumatic brain injury and stroke using the Canadian Occupational Performance Measure. Am. J. Occup. Ther..

[48] Yang S.-Y., Lin C.-Y., Lee Y.-C., Chang J.-H. (2017). The Canadian occupational performance measure for patients with stroke: A systematic review. J. Phys. Ther. Sci..

[49] Cup E.H., Scholte op Reimer W., Thijssen M.C., Kuyk-Minis M. (2003). Reliability and validity of the Canadian Occupational Performance Measure in stroke patients. Clin. Rehabil..

[50] Martini R., Rios J., Polatajko H., Wolf T., McEwen S. (2015). The performance quality rating scale (PQRS): Reliability, convergent validity, and internal responsiveness for two scoring systems. Disabil. Rehabil..

[51] Kean J., Malec J.F., Altman I.M., Swick S. (2011). Rasch measurement analysis of the Mayo-Portland Adaptability Inventory (MPAI-4) in a community-based rehabilitation sample. J. Neurotrauma.

[52] Malec J.F., Lezak M.D. (2008). Manual for the Mayo-Portland Adaptability Inventory (MPAI-4) for adults, children and adolescents. http://www.tbims.org/combi/mpai/manual.

[53] Guerrette M.-C., McKerral M. (2021). Validation of the Mayo-Portland Adaptability Inventory-4 (MPAI-4) and reference norms in a French-Canadian population with traumatic brain injury receiving rehabilitation. Disabil. Rehabil..

[54] Fortune D.G., Walsh R.S., Waldron B., McGrath C., Harte M., Casey S., McClean B. (2015). Changes in aspects of social functioning depend upon prior changes in neurodisability in people with acquired brain injury undergoing post-acute neurorehabilitation. Front. Psychol..

[55] Malec J.F., Kean J., Altman I.M., Swick S. (2012). Mayo-Portland Adaptability Inventory: Comparing psychometrics in cerebrovascular accident to traumatic brain injury. Arch. Phys. Med. Rehabil..

[56] Eicher V., Murphy M.P., Murphy T.F., Malec J.F. (2012). Progress assessed with the Mayo-Portland Adaptability Inventory in 604 participants in 4 types of post–inpatient rehabilitation brain injury programs. Arch. Phys. Med. Rehabil..

[57] Altman I.M., Swick S., Parrot D., Malec J.F. (2010). Effectiveness of community-based rehabilitation after traumatic brain injury for 489 program completers compared with those precipitously discharged. Arch. Phys. Med. Rehabil..

[58] Wilson B.A., Evans J.J., Emslie H., Alderman N., Burgess P. (1998). The development of an ecologically valid test for assessing patients with a dysexecutive syndrome. Neuropsychol. Rehabil..

[59] Boelen D.H., Spikman J.M., Rietveld A.C., Fasotti L. (2009). Executive dysfunction in chronic brain-injured patients: Assessment in outpatient rehabilitation. Neuropsychol. Rehabil..

[60] Azouvi P., Vallat-Azouvi C., Millox V., Darnoux E., Ghout I., Azerad S., Ruet A., Bayen E., Pradat-Diehl P., Aegerter P. (2015). Ecological validity of the dysexecutive questionnaire: Results from the PariS-TBI study. Neuropsychol. Rehabil..

[61] Burgess P.W., Alderman N., Evans J., Emslie H., Wilson B.A. (1998). The ecological validity of tests of executive function. J. Int. Neuropsychol. Soc..

[62] Chen G., Gully S.M., Eden D. (2001). Validation of a new general self-efficacy scale. Organ. Res. Methods.

[63] Scherbaum C.A., Cohen-Charash Y., Kern M.J. (2006). Measuring General Self-Efficacy: A comparison of three measures using item response theory. Educ. Psychol. Meas..

[64] Jacobs J.M., Hammerman-Rozenberg A., Stessman J. (2018). Frequency of leaving the house and mortality from Age 70 to 95. J. Am. Geriatr. Soc..

[65] Ornstein K.A., Leff B., Covinsky K.E., Ritchie C.S., Federman A.D., Roberts L., Kelley A.S., Siu A.L., Szanton S.L. (2015). Epidemiology of the homebound population in the United States. JAMA Intern. Med..

[66] Bedard M., Molloy D.W., Squire L., Dubois S., Lever J.A., O’Donnell M. (2001). The Zarit Burden Interview: A new short version and screening version. Gerontologist.

[67] Van Durme T., Macq J., Jeanmart C., Gobert M. (2012). Tools for measuring the impact of informal caregiving of the elderly: A literature review. Int. J. Nurs. Stud..

[68] Iecovich E. (2012). Psychometric properties of the Hebrew version of the Zarit Caregiver Burden Scale short version. Aging Ment. Health.

[69] Higginson I.J., Gao W., Jackson D., Murray J., Harding R. (2010). Short-form Zarit Caregiver Burden Interviews were valid in advanced conditions. J. Clin. Epidemiol..

[70] Fritz C.O., Morris P.E., Richler J.J. (2012). Effect size estimates: Current use, calculations, and interpretation. J. Exp. Psychol. Gen..

[71] Cohen J. (1992). Quantitative methods in psychology: A power primer. Psychol. Bull..

[72] Wu W., Gu F., Fukui S. (2021). Combining proration and full information maximum likelihood in handling missing data in Likert scale items: A hybrid approach. Behav. Res. Methods.

[73] Salkind N. (2010). Last observation carried forward. Encyclopedia of Research Design.

[74] McEwen S.E., Polatajko H.J., Huijbregts M.P., Ryan J.D. (2009). Exploring a cognitive-based treatment approach to improve motor-based skill performance in chronic stroke: Results of three single case experiments. Brain Inj..

[75] Henshaw E., Polatajko H., McEwen S., Ryan J.D., Baum C.M. (2011). Cognitive approach to improving participation after stroke: Two case studies. Am. J. Occup. Ther..

[76] Song C.-S., Lee O.-N., Woo H.-S. (2019). Cognitive strategy on upper extremity function for stroke: A randomized controlled trials. Restor. Neurol. Neurosci..

[77] Levack W.M., Dean S.G., Siegert R.J., Mcpherson K.M. (2006). Purposes and mechanisms of goal planning in rehabilitation: The need for a critical distinction. Disabil. Rehabil..

[78] Sugavanam T., Mead G., Bulley C., Donaghy M., Van Wijck F. (2013). The effects and experiences of goal setting in stroke rehabilitation—A systematic review. Disabil. Rehabil..

[79] Houldin A., McEwen S.E., Howell M.W., Polatajko H.J. (2018). The Cognitive Orientation to Daily Occupational Performance approach and transfer: A scoping review. OTJR: Occup. Particip. Health.

[80] McEwen S.E., Houldin A., Dawson D.R., McEwen S.E., Polatajko H.J. (2017). Generalization and Transfer in the CO-OP Approach. Cognitive Orientation to Daily Occupational Performance in Occupational Therapy: Using the CO-OP ApproachTM to Enable Participation Across the Life Span.

[81] McEwen S.E., Polatajko H.J., Davis J.A., Huijbregts M., Ryan J.D. (2010). ‘There’s a real plan here, and I am responsible for that plan’: Participant experiences with a novel cognitive-based treatment approach for adults living with chronic stroke. Disabil. Rehabil..

[82] Rotenberg-Shpigelman S., Erez A.B.-H., Nahaloni I., Maeir A. (2012). Neurofunctional treatment targeting participation among chronic stroke survivors: A pilot randomised controlled study. Neuropsychol. Rehabil..

[83] Chumbler N.R., Quigley P., Li X., Morey M., Rose D., Sanford J., Griffiths P., Hoenig H. (2012). Effects of telerehabilitation on physical function and disability for stroke patients: A randomized, controlled trial. Stroke.

[84] Polatajko H.J., McEwen S.E., Ryan J.D., Baum C.M. (2012). Pilot randomized controlled trial investigating cognitive strategy use to improve goal performance after stroke. Am. J. Occup. Ther..

[85] Falvey J.R., Cohen A.B., O’Leary J.R., Leo-Summers L., Murphy T.E., Ferrante L.E. (2021). Association of social isolation with disability burden and 1-year mortality among older adults with Ccritical illness. JAMA Intern. Med..

[86] Ankuda C.K., Leff B., Ritchie C.S., Siu A.L., Ornstein K.A. (2021). Association of the COVID-19 pandemic with the prevalence of homebound older adults in the United States, 2011–2020. JAMA Intern. Med..

[87] Moo L.R., Schwartz A.W. (2021). The urgent need for rigorous studies of telehealth for older adults who are homebound. JAMA Netw. Open.

[88] Skidmore E.R., McEwen S.E., Green D., van den Houten J., Dawson D.R., Polatajko H.J., Dawson D.R., McEwen S.E., Polatajko H.J. (2017). Essential Elements and Key Features of the CO-OP Approach. Cognitive Orientation to Daily Occupational Performance in Occupational Therapy: Using the CO-OP ApproachTM to Enable Participation Across the Life Span.

[89] Martini R., Savard J. (2021). Cognitive Orientation to Daily Occupational Performance (CO-OP): 1-week group intervention with children referred for motor coordination difficulties. Open J. Occup. Ther..

